# Literary Notes

**Published:** 1896-09

**Authors:** 


### Literary Notes.

Lea Brothers & Co., medical publishers, Philadelphia, announce that they will issue, early in September, a revised edition of Gray's Anatomy. Every page of this new edition has received the careful scrutiny of a corps of eminent American anatomists, who have made such changes as seemed to appear necessary to represent advances in anatomical knowledge and improved methods of teaching since the thirteenth edition of Gray was published. Students and teachers will be eager to obtain the forthcoming book. The price will be retained at the previous figure.

Medical Press Bureau.—At the solicitation of a number of prominent medical publishers, Mr. Charles Wood Fassett has decided to conduct a medical press bureau at the prominent medical association meetings held in this and foreign countries. The plan will be inaugurated at the meeting of the Mississippi Valley Medical Society, in St. Paul, September 15, 1896, and in brief is as follows: Ample space is to be reserved in the medical exhibit hall for a library and reading room, for the use of the visiting doctors, in which will be placed the principal medical journals of the country, and where subscriptions and advertising will be received, sample copies and advertising matter distributed. A neat catalogue will be issued, giving full description of each periodical, together with its subscription price and special features. These will be distributed at headquarters, in the principal hotels, and in all rooms where exhibits are placed, thus reaching not only all the physicians in attendance, but the advertisers as well. Besides this, a table will be reserved in the hotel writing rooms, where a number of copies of each journal will be placed at the disposal of the delegates, for evening reading and consulting.

Mr. Fassett has been a regular attendant at the national and state societies for a number of years, in the interest of journalism, and is enthusiastic as to the benefits accruing to a proper representation at these annual meetings. Doctors and advertising patrons admire enterprise and patriotism when evinced in a proper manner. It goes without saying that the highest compliment you can pay a medical society or an advertiser, is to place your journal where the society members can examine it while they are "off duty" and outside the confines of their office.

And yet we see but very few journals represented at even the largest meetings. And why? Simply because of the expense of sending and maintaining a representative. Especially is this true of those publishers who are located in remote portions of the country. Consequently they are seldom heard of except at local meetings. The plan for a coöperative representation, as outlined above, is both feasible and desirable, and in the hands of so experienced a manager as Mr. Fassett cannot fail to be a success. It will advance the interests of the entire medical press of America, and will impress every patron in a most favorable manner. A small fee will be charged, to cover expenses, and the membership will be limited. We advise every publisher to communicate at once with Mr. Fassett and obtain details of the plan.—*Bulletin of the American Publishers Association.*

